# Surgical Management and Reconstruction of Dedifferentiated Chondrosarcoma in the Proximal Femur: A Case Report

**DOI:** 10.7759/cureus.63184

**Published:** 2024-06-26

**Authors:** El Mehdi Lahrach, Abdeloihab Jaafar, Najib Al Idrissi

**Affiliations:** 1 Orthopedics and Traumatology, Cheikh Khalifa International University Hospital, Mohammed VI University of Health Sciences (UM6SS), Casablanca, MAR; 2 School of Medicine, Laboratory of Genomics, Epigenetics, Personalized and Predictive Medicine, Mohammed VI University of Health Sciences (UM6SS), Casablanca, MAR

**Keywords:** bone tumor resection, proximal femur, oncological surgery, massive prosthesis reconstruction, dedifferentiated chondrosarcoma

## Abstract

A primary malignant bone tumor, or more commonly, metastasis, can occur in the proximal femur. Surgical treatment can have palliative or curative purposes. In the case of the latter, it involves two stages: resection of the tumor, which aims to address the cancer, and reconstruction of the bone and soft tissue, which aims to restore function. It is important for the excision to be wide with adequate resection margins in the soft tissue, particularly when the goal is curative treatment. Typically, surgery involves excision and reconstruction to ensure good mechanical stability. Reconstruction can be done using different methods, such as a composite prosthesis or a massive prosthesis, which may be modular or custom-made. Joint reconstruction options include hemiarthroplasty, intermediate prosthesis, or, in some cases, total hip replacement.

## Introduction

Primary malignant bone tumors in the proximal femur are infrequent, comprising approximately 9% to 10% of cases, with chondrosarcomas being the most prevalent type. Nonetheless, the proximal femur is subject to a high occurrence of bone metastasis from osteophilic cancer, resembling spinal metastases [1]. The treatment for proximal femur tumors necessitates a two-part strategy incorporating tumor resection for oncological reasons and bone and soft tissue reconstruction for mechanical purposes [1-3] Our report details a case of dedifferentiated chondrosarcoma located in the upper extremity of the femur.

## Case presentation

**Figure 1:**
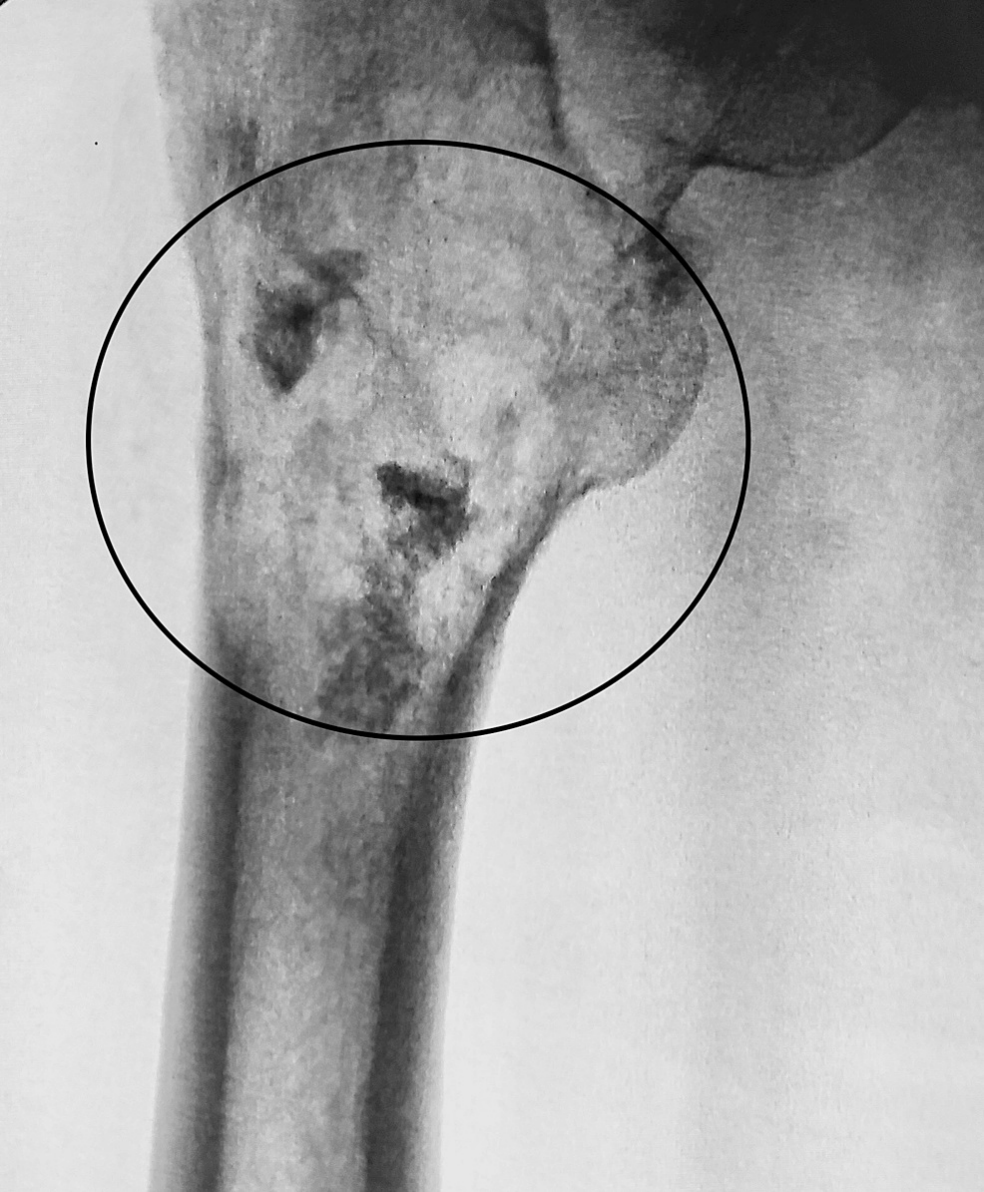
Intraoperative image displaying an osteolytic appearance indicating the pathological nature of the lesion.

In May 2021, a 73-year-old patient sought medical attention for a painful mass located on the upper, outer part of their right thigh. Standard radiography revealed the presence of bone destruction at the top of the right femur. In June 2021, computed tomography and an MRI specifically focused on the right femur indicated a low-density signal on T1 and a high-density signal on T2. These findings suggested the presence of an expanding lesion in the upper metaphyseal-diaphyseal region of the right femur. The lesion extends over a 15 cm area, has caused cortical rupture, and has spread to the surrounding soft tissues (Figure 1).

**Figure 2:**
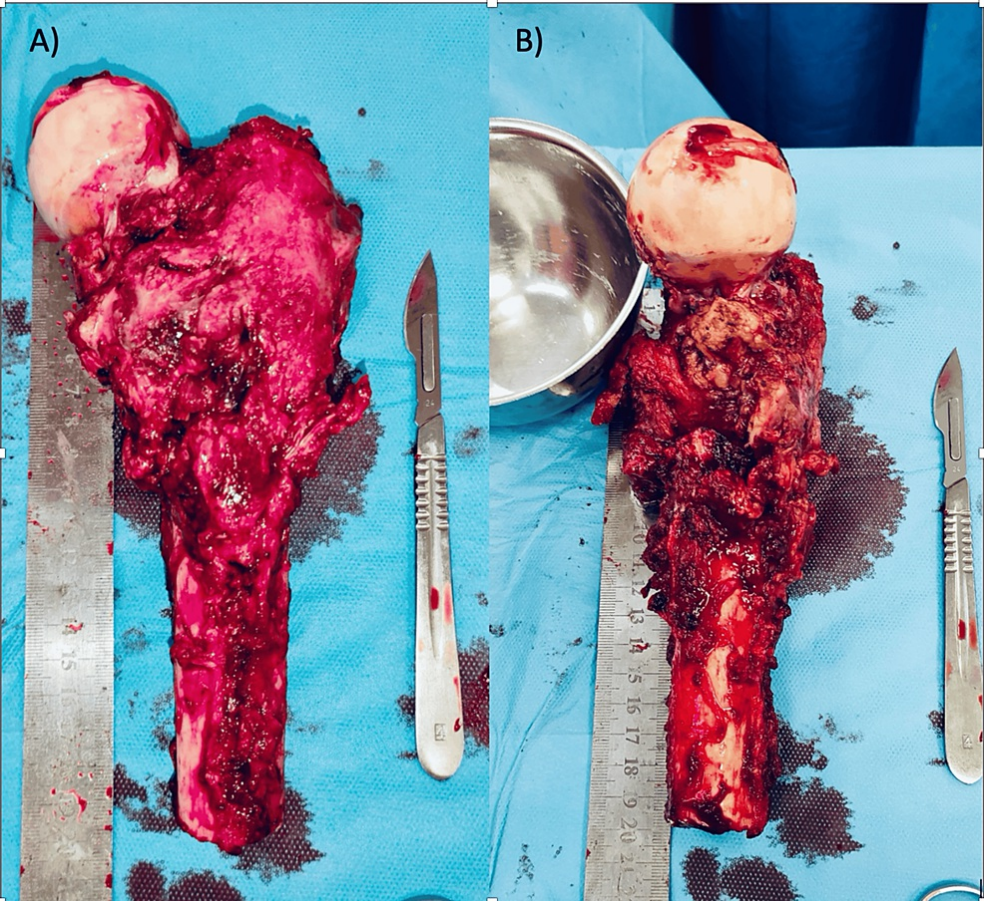
Removal of the tumor lesion located at the proximal end of the right femur.

In August 2021, the patient had surgical biopsies on the right femur's lateral aspect, which revealed a poorly differentiated tumor process in all samples. A positron emission tomography (PET) extension balance tomography using 18F-FDG did not show any other secondary localization. The patient's case was reviewed in a multidisciplinary consultation meeting, and it was recommended to undergo surgical treatment in the form of a wide extralesional excision 15 cm from the right trochanteric region (Figure 2).

**Figure 3:**
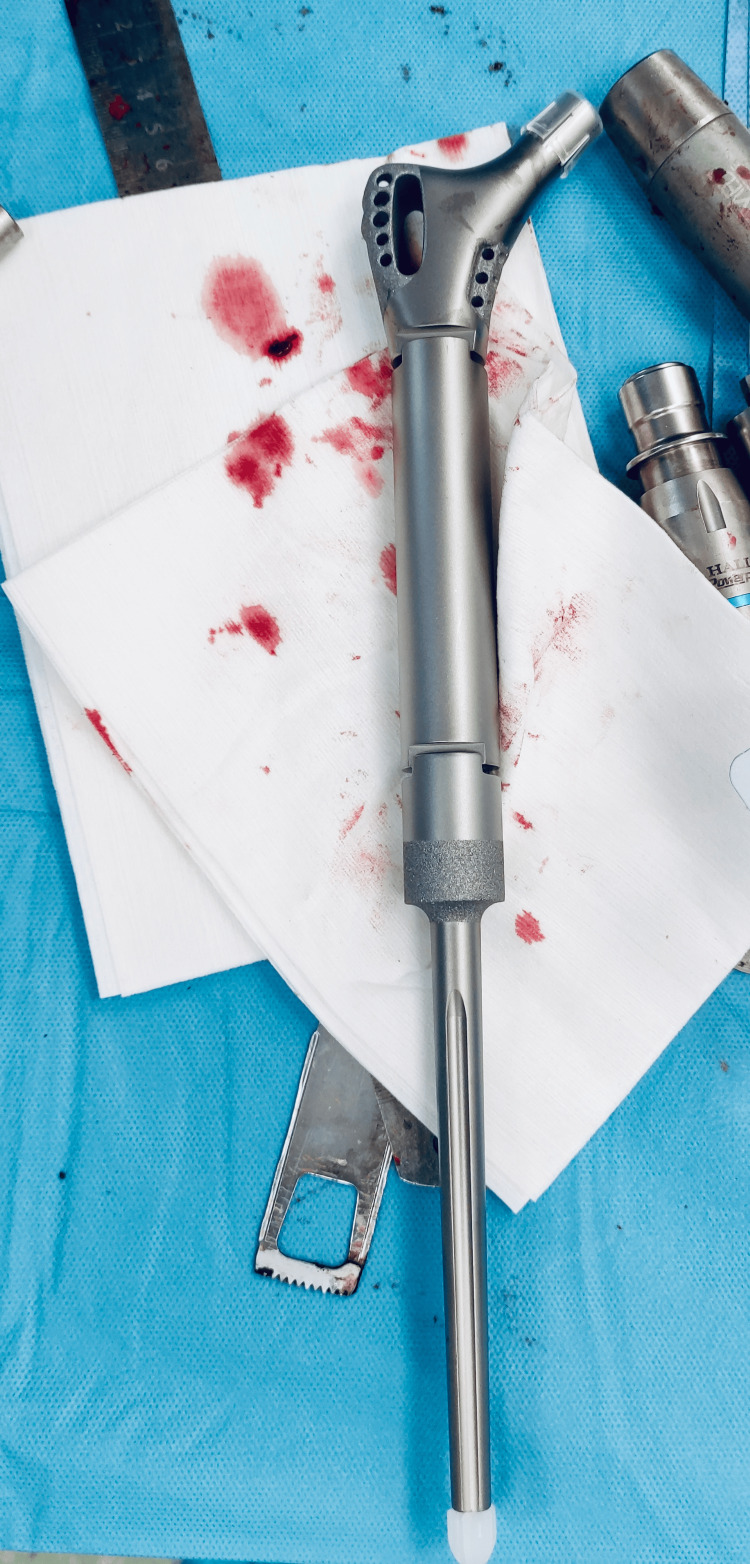
The intraoperative process of preparing the large prosthesis for the right femur.

The upper portion of the right femur was reconstructed using a large prosthesis (Figure 3).

**Figure 4:**
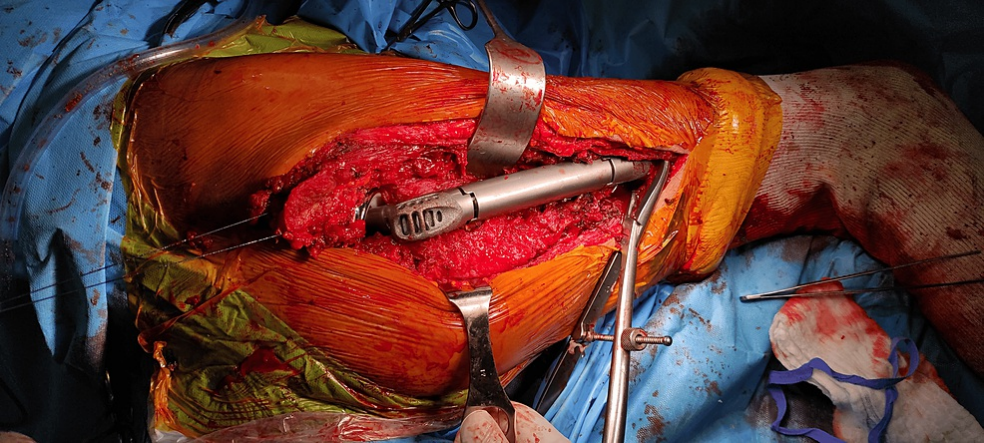
Positioning of the significant prosthesis at the top of the right femur, demonstrating the reattachment of the tendons of the iliac and gluteal medius psoas muscles.

The coxofemoral joint was repaired in multiple stages using an intermediate prosthesis. The trabecular region was reconstructed with a 5 cm stage, followed by two femoral stages of equal length. A bipolar spherical head of 50/28 mm was used. The prosthesis was extended with a 135 mm rod with a 12 mm diameter, which was cemented while maintaining the trochanteric insertion of the abductor apparatus (Figure 4).

**Figure 5:**
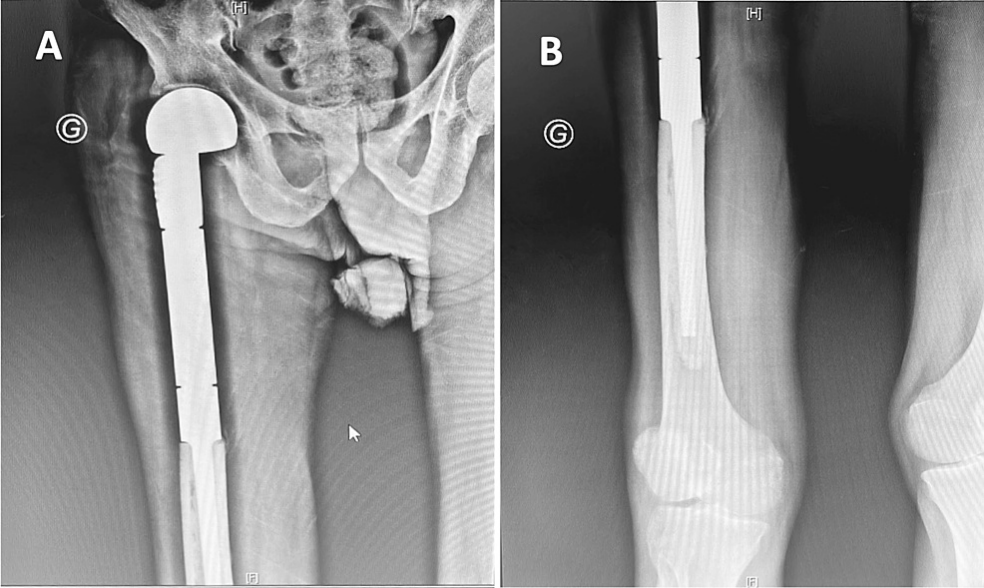
The postoperative X-ray shows (A) the proximal femur prosthesis and (B) its insertion into the distal medullary canal.

The patient showed no signs of neurological impairment. Upon completion of the final histopathological examination and immunohistochemistry analysis, it was determined that there was a tumor mass measuring 6.5 × 3 cm, indicating the presence of a grade 2 dedifferentiated chondrosarcoma. The tumor was successfully removed with clear margins (R0), and further treatment involving chemotherapy and radiotherapy was recommended. After nine months, the patient was able to walk intermittently with crutches and had achieved 90 degrees of hip flexion. Subsequent clinical monitoring did not reveal any indications of local or metastatic recurrence (Figure 5).

## Discussion

Chondrosarcoma, the second most common primary malignant bone tumor following osteosarcoma, accounts for 25% of all primary malignant bone tumors and shows a slight male predominance. Its annual incidence is estimated at 1/200,000 [4] and typically occurs in individuals aged 40 to 70, with half of patients being over 40 years old. About 10% of chondrosarcomas develop on pre-existing bone lesions, including benign cartilage tumors such as osteochondroma or chondroma. These tumors appear on bones with enchondral ossification, indicating a potential link between the development of tumor cells and the chondral cells of the growth plate [4].

Dedifferentiated chondrosarcomas are a subclass of low-grade chondrosarcomas with a high-grade malignancy and distinct histological features. They are primarily identified by their histology and have a poor prognosis. These tumors make up 10 to 17% of chondrosarcomas and are typically discovered at the same average age as conventional chondrosarcomas. However, they progress much more rapidly. Dedifferentiation can occur either initially or during a local recurrence of a previously treated low-grade chondrosarcoma. Pain is the most common presenting symptom in the majority of cases [5].

The occurrence of a pathological fracture, which is relatively rare in a chondrosarcoma, should be carefully noted. Such fractures happen in 13 to 25% of cases. The laboratory testing results typically show no abnormalities, with the absence of any inflammatory syndrome. Approximately 80% of patients with chondrosarcoma experience carbohydrate intolerance during induced hyperglycemia, although this has no practical clinical significance. The areas most commonly affected are the coxal bone (approximately one third) and the proximal femur (about one quarter) [6].

The radiological diagnosis is frequently highly suspected on standard X-rays. It is feasible to differentiate between central forms, periosteal forms, and surface chondrosarcomas that arise during the malignant transformation of an osteochondroma. The CT scan allows for a more precise assessment of the resorption and potential rupture of the cortex. Furthermore, intramatrix calcifications are also readily visualized with this examination [7].

MRI is of specific significance for radiolucent tumors and for evaluating the extent of locoregional extension. These tumors appear hyperintense in T2 and have an intermediate signal in T1. In cases of well-differentiated tumors, it is feasible to observe the cartilage lobules. The absence of bone marrow entirely between these lobules strongly suggests the malignant nature of the cartilage lesion [8].

From a histological perspective, it is common to observe a high-grade non-cartilaginous sarcoma adjacent to a low-grade chondrosarcoma. This low-grade chondrosarcoma is frequently seen as a high-grade osteosarcoma or undifferentiated spindle cell sarcoma, with occasional expression of muscle markers. This suggests a progression rather than tumor dedifferentiation [9].

The surgical treatment of chondrosarcoma in the proximal femur may be either palliative or curative. In the case of the latter, it involves two stages: the removal of the tumor and the reconstruction of bone and soft tissue for functional purposes. It is essential that the surgical excision is wide, with adequate margins of resection. Reconstruction may involve the use of a composite prosthesis, which consists of a femoral rod encased in a bone or osteotendinous allograft, or a modular or customized massive prosthesis. For small lesions limited to the epiphysis and cervix, a standard or even long femoral shaft may be used. Reconstruction of the coxofemoral joint can be achieved through an intermediate or total hip replacement. The first step in excision surgery is the surgical biopsy, which should be performed by the attending surgeon. The location of the biopsy scar should take into consideration the initial route of the future resection to be conducted en bloc with the excision piece. The planning of the resection is based on an MRI, which should be carried out no later than four weeks before the surgical procedure [10].

The wide excision of the proximal femur commences with positioning the patient in a lateral position, supported by pubic and sacral supports, similar to a standard prosthesis. The approach focuses on the external face of the great trochanter, permitting anterior dissection on the limb in external rotation and posterior on the limb in internal rotation. It extends to the outer surface of the diaphysis based on the planned distal resection, and proximal to the outer surface of the gluteal fan at 8 to 10 cm. The sciatic nerve is then identified and safeguarded by the eversion of these muscles. Releasing the distal tendon of the gluteus maximus muscle of the femur is often necessary. If the great trochanter is invaded, it is recommended to cut the tendons of the gluteus middle and gluteus minimus muscles. Dissection proceeds to the iliopsoas muscle, which is cut at a distance from its insertion, and more distally to the pectinate and adductor muscles. The external collateral branches of the femoral artery, which are the branches of the anterior circumflex, are ligated as required. The distal section is determined by measuring the preoperative planned distance from the top of the great trochanter; for larger resections, it is sometimes easier to measure the height of the distal osteotomy from the joint space of the knee. At the distal section area, the deep part of the quadriceps is cut to the bone to expose the diaphysis and perform the bone cut. The hamstrings are relaxed at the back. The medial dissection enables the extraction of the part. The distal end of the piece is placed on a davier, and the adductors are then released internally. The round ligament is cut flush with the acetabula, and the piece is removed. Careful hemostasis is performed, and reconstruction can commence [11].

In contrast to carcinological excision, intralesional excision does not necessitate the observance of margins. It is crucial to always preserve the trochanteric insertion of the abductor apparatus because of its functional significance [12].

After resection of the proximal end of the femur, reconstruction can be accomplished through a variety of techniques. This includes the use of composite prosthesis with allografts of nonirradiated proximal femur, pure bone, or osteotendinous components involving the distal tendon of the gluteus medius and the psoas. Another method involves the use of a massive prosthesis, such as a femoral prosthesis with an enlarged diaphyseal part to accommodate for longer resections. After preparing the remaining femoral diaphysis, options include cementing with hard friction, standard cementing, or using an impacted part without cement. A modular test implant is placed with a diaphyseal piece corresponding to the height of resection, and the height and anteversion are checked before placing the final implant. It is recommended to use a small epiphysis to reduce offset and minimize the work of gluteal muscles [13].

The hip can be reconstructed and stabilized through the use of either an intermediate or total prosthesis. In cases where there is no tumor involvement in the cotyloid, the preferred option is to first utilize hemiarthroplasty with an intermediate prosthesis.

## Conclusions

Chondrosarcoma, a type of tumor that is not very responsive to chemotherapy, required a necessary excision in a healthy area, making the surgical indication undeniable. The final pathological analysis showed that the margins of excision were satisfactory, and the surrounding soft tissue boundaries were also healthy. Since allografts and bone banks are not available in Morocco, reconstruction using a massive prosthesis was preferred over a composite prosthesis and hemiarthroplasty in cases where cotyloid involvement was not present. The latter option has a 96% survival rate at 15 years and a very low revision rate for totalization, as well as a lower dislocation rate compared to total dentures. 
